# Oral acantholytic squamous cell carcinoma shares clinical and histological features with angiosarcoma

**DOI:** 10.1186/1746-160X-4-17

**Published:** 2008-07-31

**Authors:** Oliver Driemel, Urs DA Müller-Richter, Samer G Hakim, Richard Bauer, Alexander Berndt, Johannes Kleinheinz, Torsten E Reichert, Hartwig Kosmehl

**Affiliations:** 1 Department of Oral and Maxillofacial Surgery, University of Regensburg, Franz-Josef-Strauss-Allee 11, 93053, Regensburg, Germany; 2 Department of Oral and Maxillofacial Plastic Surgery, University of Würzburg, Pleicherwall 2, 97070, Würzburg, Germany; 3 Department of Maxillofacial Surgery, University Medical Center Schleswig-Holstein, Campus Lübeck, Ratzeburger Allee 160, 23538, Lübeck, Germany; 4 Department of Pathology, University of Jena, Ziegelmühlenweg 1, 07740, Jena, Germany; 5 Department of Maxillofacial Surgery, University of Münster, Waldeyerstraße 30, 48149, Münster, Germany; 6 Institute of Pathology, HELIOS Hospital Erfurt, Nordhäuser Strasse 74, 99089, Erfurt, Germany

## Abstract

**Background:**

acantholytic squamous cell carcinomas (ASCC) and intraoral angiosarcoma share similar histopathological features. Aim of this study was to find marker for a clear distinction.

**Methods:**

Four oral acantholytic squamous cell carcinomas and one intraoral angiosarcoma are used to compare the eruptive intraoral growth-pattern, age-peak, unfavourable prognosis and slit-like intratumorous spaces in common histological staining as identical clinical and histopathological features. Immunohistochemical staining for pancytokeratin, cytokeratin, collagen type IV, γ2-chain of laminin-5, endothelial differentiation marker CD31 and CD34, F VIII-associated antigen, Ki 67-antigen, β-catenin, E-cadherin, α-smooth-muscle-actin and Fli-1 were done.

**Results:**

Cytokeratin-immunoreactive cells can be identified in both lesions. The large vascularization of ASCC complicates the interpretation of vascular differential markers being characteristic for angiosarcoma. Loss of cell-cell-adhesion, monitored by loss of E-cadherin and β-catenin membrane-staining, are indetified as reasons for massive expression of invasion-factor ln-5 in ASCC and considered responsible for unfavourable prognosis of ASCC. Expression of Fli-1 in angiosarcoma and cellular immunoreaction for ln-5 in ASCC are worked out as distinguishing features of both entities.

**Conclusion:**

Fli-1 in angiosarcoma and ln-5 in ASCC are distinguishing features.

## Background

Both oral angiosarcoma and oral acantholytic squamous cell carcinoma (ASCC) are well-defined entities. The WHO classification of tumours describes angiosarcoma as a malignant tumour consisting of cells recapitulating variably the morphological and functional features of normal endothelium, ICD-O code 9120/3 [1-3]. ASCC (synonyms: acantholytic squamous cell carcinoma, adenoid squamous carcinoma, pseudoglandular squamous cell carcinoma, squamous cell carcinoma with glandlike (adenoid) features, angiosarcoma-like squamous cell carcinoma, adenoacanthoma, pseudovascular adenoid squamous cell carcinoma, pseudoangiosarcomatous carcinoma) is characterized as a squamous cell carcinoma containing pseudo-glandular spaces or lumina, ICD-O code 8075/3 [4,5].

Although angiosarcoma (malignant soft tissue tumour) and ASCC present conceptually complete different tumour entities their histological features are similar and defined by intratumorous spaces. Interestingly both tumour entities show comparable clinical appearance in the oral cavity. The peak incidence of angiosarcoma is the 7^th ^decade [6] and the peak incidence of the oral ASCC is the 6^th ^decade [7]. Macroscopically both entities express in oral cavity fast growing, eruptive lesions and have poor prognosis [8,9]. Like all oral squamous cell carcinomas ASCC show male predilection of 1 to 3.5 whereas no sex predilection of oral angiosarcoma is known.

Common and different aspects of oral angiosarcoma and ASCC will be worked out for the correct differential diagnosis. The cellbiological background explaining the peculiar pseudovascular appearance of ASCC is elucidated.

## Methods

### Clinical features

A 63-year-old male patient presented with a polypous, superficial ulcerated, 1.5 × 1 × 1 cm^3 ^large mass at the alveolar ridge. A biopsy was taken and the histological diagnosis of an angiosarcoma was established. Metastases developed in pleurae (cytologically verified) and ileum one month after diagnosis of the primary oral lesion. Although an ileum segment resection was carried out the patient died of angiosarcoma induced intestinal bleeding two months after initial diagnosis.

The clinical data of the ASCC are summarized in table 1.

**Table 1:** Clinical features of patients with acantholytic squamous cell carcinoma (ASCC).

**Case**	**Age**	**Gender**	**Site**	**TNM**
1	58	f	right alveolar ridge of the lower jaw	pT4 pN0 cM0 L1 V1
2	57	m	right border of the tongue	cT4
3	68	m	tongue	cT1
4	50	m	floor of the mouth	pT4a pN1 cM0 L1 V0

f: female, m: male

**Figure 1:**
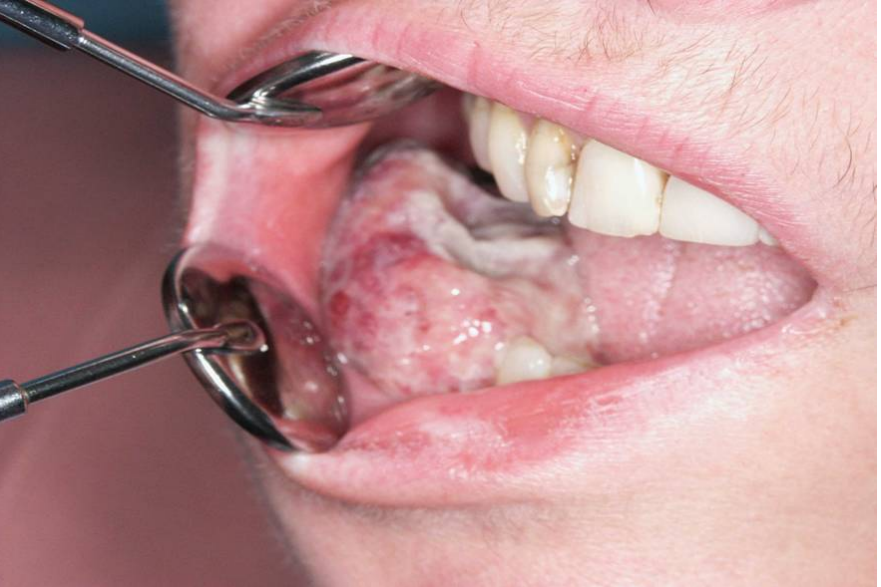
**Exophytic growth of an oral acantholytic squamous cell carcinoma on the alveolar ridge of the lower jaw**.

With the exception of case 3 which represents a metachronical ASCC after multimodal therapy of a hypopharyngeal squamous cell carcinoma all ASCC were diagnosed in an advanced stage. Case 1 developed regional lymph node and distant metastases during adjuvant radiotherapy (Figure. 1).

### Methods

For comparative analysis the tissue of the diagnostic tumour biopsies was fixed in 4.0% buffered formalin and embedded in paraffin. The slides were stained with H&E, PAS, Goldner's trichrome staining and Gömöri.

### Immunohistochemistry

Primary antibodies applied in the study: pancytokeratin (clones AE1/AE3, Dako, Denmark) dilution 1:20, cytokeratin (clone MNF-116, Dako, Denmark) dilution 1:200, collagen type IV (clone C22, Dako, Denmark) dilution 1:200, γ2-chain of laminin-5 (clone D4B5, Chemicon, USA) dilution 1:10000, endothelial differentiation marker CD31 (clone JC/70A, Dako, Denmark) dilution 1:100, CD34 (clone QBEND 10, Immunotech, France) dilution 1:500, F VIII-associated antigen (clone F 8/86, Dako, Denmark) dilution 1:200, Ki 67-antigen (clone MIB-1, Dako, Denmark) dilution 1:1000, β-catenin (clone 17 C 2, Novacastra, U.K.) 1:200, E-cadherin (clone 4A2C7, Zymed, USA) dilution 1:75, α-smooth-muscle-actin (clone 1A4, Dako, Denmark) dilution 1:400, Fli-1 (polyklonal, Zymed, USA). Primary antibodies were detected using the streptavidin-biotin-alkaline phosphatase-technique (ChemMate, Dako, Denmark). The immunohistochemical procedure was carried out at autostainer plus according to the manufactures' protocol (Dako, Denmark).

## Results

### Histopathologic findings

**Figure 2:**
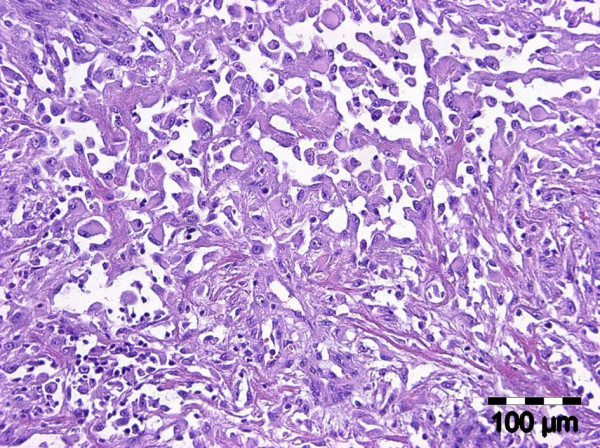
**Oral acantholytic squamous cell carcinoma: capillary and papillary growth pattern (H&E, ×150)**.

**Figure 3:**
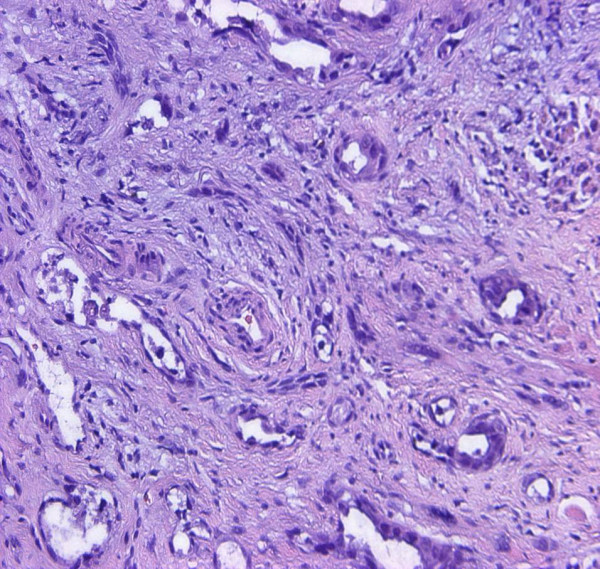
Oral acantholytic squamous cell carcinoma: venular/glandular-like pattern (H&E, ×150).

The diagnostic biopsies of both entities showed a superficial necrotic zone due to ulceration. The tumour cells were large and showed a polygonal to epitheloid shape. There was a highly pathologic nucleus-cytoplasm-ratio. Prominent nucleoli were a continuous feature. The tumour cells of both entities contained a fine granular PAS-positive material within the cytoplasm. Both lesions were characterized by slit-like intratumorous spaces or papillary and pseudopapillary projections (Figure. 2). In case 3, additionally to the slit-like tumourous spaces a venular- or glandular-like pattern was formed (Figure. 3). The Gömöri staining revealed a discontinuous staining in the basement membrane region at the tumour cell stroma interface. In more solid tumour areas the Gömöri staining demonstrated an acinar or trabecular growth pattern. A dysplastic covering oral mucosa could not be evidenced due to ulceration. Only in one ASCC, dyskeratosis could be evidenced in serial sections. Hemorrhagic areas were found in angiosarcoma as well as in ASCC.

### Immunohistochemical findings

**Figure 4:**
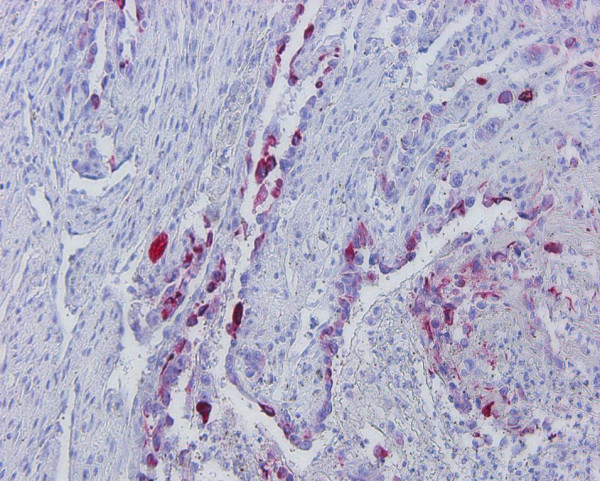
**Oral angiosarcoma: immunohistochemical demonstration of the epithelial intermediate filament protein cytokeratin in a subset of the tumour cells (clones AE1/AE3 ×150)**.

Cytokeratin-positive tumour cells were recognized in both angiosarcoma as well as in the four ASCCs. The number of cytokeratin-positive tumour cells in angiosarcoma was lower than in ASCC (Figure. 4).

**Figure 5:**
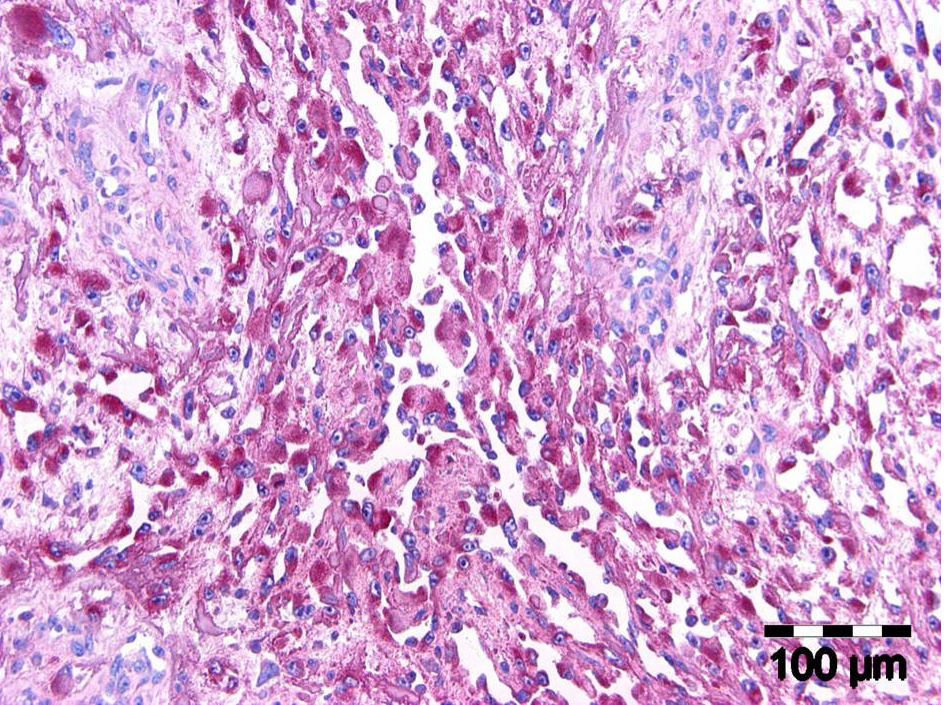
**Oral acantholytic squamous cell carcinoma: immunohistochemical demonstration of the ln-5-γ2-chain**. Note the strong immunostaining within the cytoplasm of the majority of the carcinoma cells (clone D4B5, ×150).

Ln-5-positive basement membrane region was also found in both entities. In angiosarcoma the ln-5 immunostaining of the basement membrane was regularly localized in tumour sections beneath preexisting epithelial structures. A cellular immunostaining of laminin-5 was restricted to all four ASCCs (Figure. 5). Around the slit-like intratumorous spaces a discontinuous basement membrane immunostaining was demonstrated in both entities. Moreover, in association to spindle-shaped cells between the spaces a dot-like or membranous immunostaining was visualized using antibodies against collagen type IV.

In the stroma of both entities as well as around the slit-like intratumorous spaces α-smooth-muscle-positive cells were diagnosed and often a distinction between stroma myofibroblasts or pericytes could not be made.

**Figure 6:**
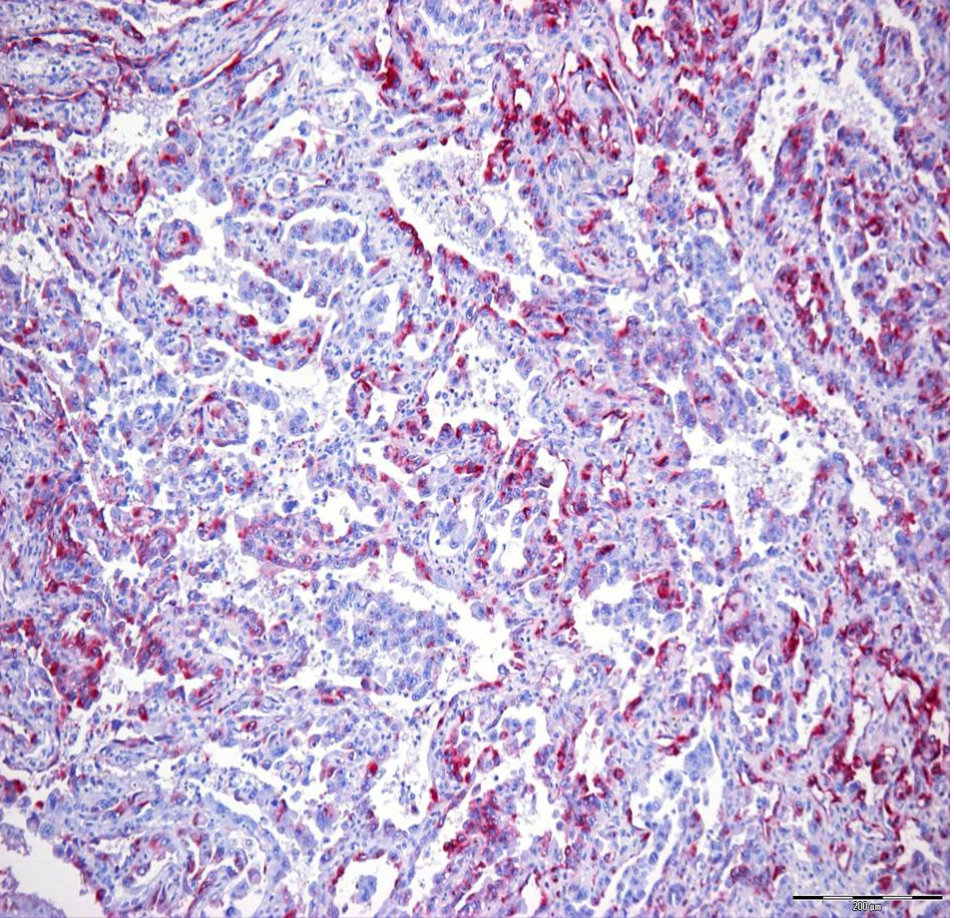
Oral angiosarcoma: immunohistochemical demonstration of factor VIII-related antigen in a subset of the tumour cells lining the vascular spaces (×150).

CD 31, CD 34 and factor VIII-associated antigen could be found in the majority of the cells of angiosarcoma (Figure. 6). The endothelial differential markers have to be interpreted carefully, because in angiosarcoma not all tumour cells are stained immunohistochemically positive and in ASCC a large vascularization characterized by positive endothelial differential markers is regularly observed.

The proliferative activity did not discriminate angiosarcoma from ASCC. The Ki 67-index reached 20%.

**Figure 7:**
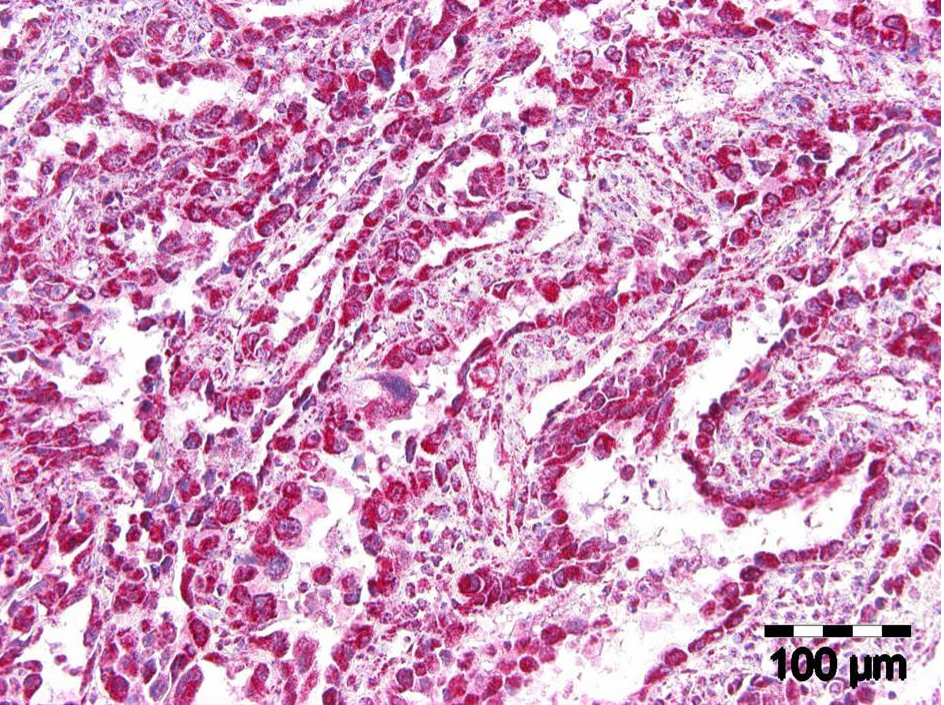
**Oral angiosarcoma: immunohistochemical demonstration of Fli-1 in a subset of the tumour cells (×150)**.

Fli-1 immunoreactivity was only recognized in angiosarcoma (Figure. 7).

E-cadherin and β-catenin were found in all four ASCCs but not in the angiosarcoma. In the majority of the tumour cells there was an E-cadherin immunostaining in cytoplasm and not at the cell membrane. Sometimes β-catenin was seen not only in the cytoplasm but also within the nucleus.

## Discussion

Several authors have already emphasized the histopathologic similarity of ASCC and angiosarcoma. [10-13]. Although the WHO defines ASCC as an original entity for a long time [4,5], there are less than thirty cases of ASCCs documented in the international literature so far [7]. Both entities may have an association to previous exposal to ionizing radiation [9,14]. To determine differential diagnosis and to differentiate ASCC from angiosarcoma an immunohistochemical typing is required regularly, because the epidermoid differentiation may be extremely masked by pseudovascular proliferation. Dyskeratoses may represent a rare pattern in ASCC. The immunohistochemical analysis has to consider on the one side that in soft tissue tumour angiosarcoma cytokeratin-positive cells may appear and on the other side that the plentiful vessels in the tumour stroma of ASCC are signed by endoepithelial differential markers, so that the classic discriminating differential markers cytokeratin, factor VIII-associated antigen and others are often difficult to be interpreted. [15-17]. The Fli-1-protein, a member of the ETS family of DNA-binding transcription factors was recently highlighted as a new vascular differentiation marker [18,19]. Although Fli-1 can be also rarely identified in carcinomas [20], ASCC is immunonegative for this marker, so that Fli-1 can be recommended to discriminate between Angiosarcoma and ASCC.

The incomplete border of pericytes represents an accepted feature for identifying differentiation disturbed neoplastic vessels of angiosarcoma. The pericytes are emphasized by α-smooth-muscle-actin [6]. However the incomplete border of pericytes in structures of angiosarcoma is not suitable for discriminating angiosarcoma versus ASCC, because in ASCC α-smooth-muscle-stromamyofibroblasts may mimic the pattern of pericytes lining discontinuously the slit-like tumour-spaces.

Interestingly in angiosarcoma ln-5 positive basal membranes were recognised. Ln-5 is a characteristic protein of epithelial basal membranes that is regularly identified in oral mucosa and in oral squamous cell carinoma [21]. It connects the basal membrane with the hemidesmosomes of epithelial cells and has not been described in mesenchymal basal membranes so far. Because in angiosarcoma in contrast to ASCC no cytoplasmatic marking as a sign of synthesis of ln-5-γ2-chain could be made out and because ln-5 was only identified in parts of angiosarcoma localized next to preexisting oral epithelia, it is suggested, that ln-5 of the new formed basal membranes of angiosarcoma comes from the neighbouring preexisting epithelial structures and has only been integrated into the new formed basal membranes of angiosarcoma.

The cytoplasmatic ln-5 detection of ASCC cells presents on the one hand a distinguishing feature between ASCC and angiosarcoma and on the other hand a tumour biological indicator of the unfavourable prognosis of ASCC.

An abundant detection of γ2-chain of ln-5 in carcinoma cells is correspondingly accepted in literature as an unfavourable prognostic pattern. The extracellular matrix protein stimulates invasion of carcinoma cells [22-24]. Hlubek and co-workers identified 2001 β-catenin as a transcription-factor of laminin-γ2-chain [25]. The membrane-localized β-catenin-E-cadherin-complex mediates the cell-cell-adhesion, that is obviously disturbed in ASCC and that is responsible for forming of the typical intercellular spaces [13,26,27]. In case of a disturbed forming of β-catenin-E-cadherin-complex at the carcinoma cell membrane β-catenin liberated from cell membrane is able to migrate into the cell nucleus, to act as a transcription-factor and to induce an overexpression of invasion-factor laminin-γ2-chain in ASCC.

The reduced cell-cell-adhesion and the extremely increased expression of laminin-γ2-chain are suggested as cell biological reasons for the extreme early distant metastasising of ASCC during therapy.

In summary angiosarcoma and ASCC do not only share identical clinical features and a similar histopathological pattern in common histological staining but also show overlaps of cytokeratin-expression and of expression of vascular differential markers. Expression of Fli-1 in angiosarcoma and cytoplasmatic immunoreaction for γ2-chain of ln-5 in ASCC are worked out as distinguishing features of both entities.

## Conflict of interests

The authors declare that they have no competing interests.

## Authors' contributions

OD acquisition of patients and study design, UMR study design, manuscript drafting, SGH acquisition of patients, RB immunostaining, AB study design, JK review and study design, TR study design, HK immunostaining, histopathological analysis.
